# Supplementary figures and images for: Glycemic Variability Impacted by SGLT2 Inhibitors and GLP 1 Agonists in Patients with Diabetes Mellitus: A Systematic Review and Meta-Analysis
Source: J Clin Med. 2021 Sep 9;10(18):4078. doi: 10.3390/jcm10184078 (PMC8470178; doi:10.3390/jcm10184078)

**Figure S1.**

**(a)**

**Regression of Std diff in means on BMI**

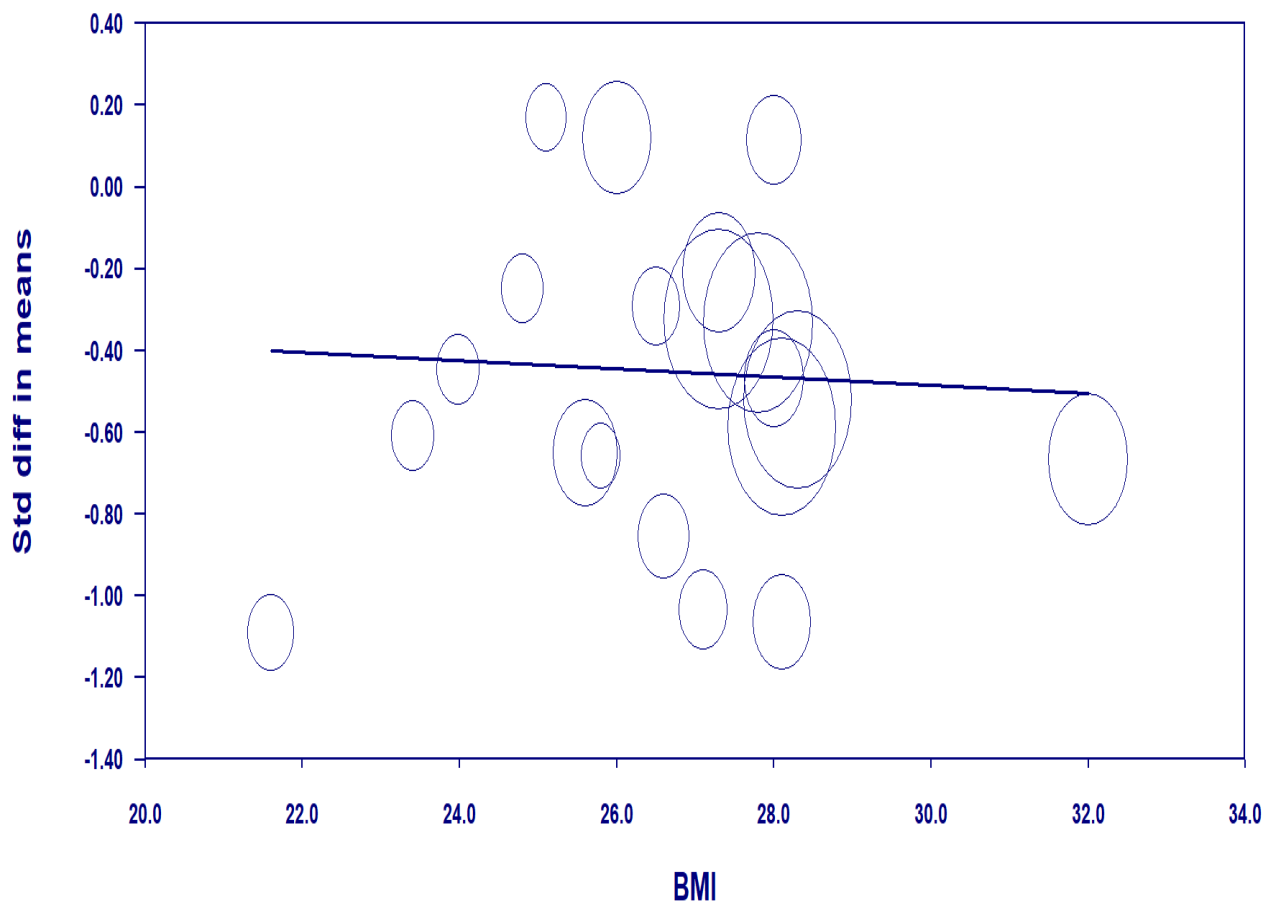

**(b)**

**Regression of Std diff in means on Age**

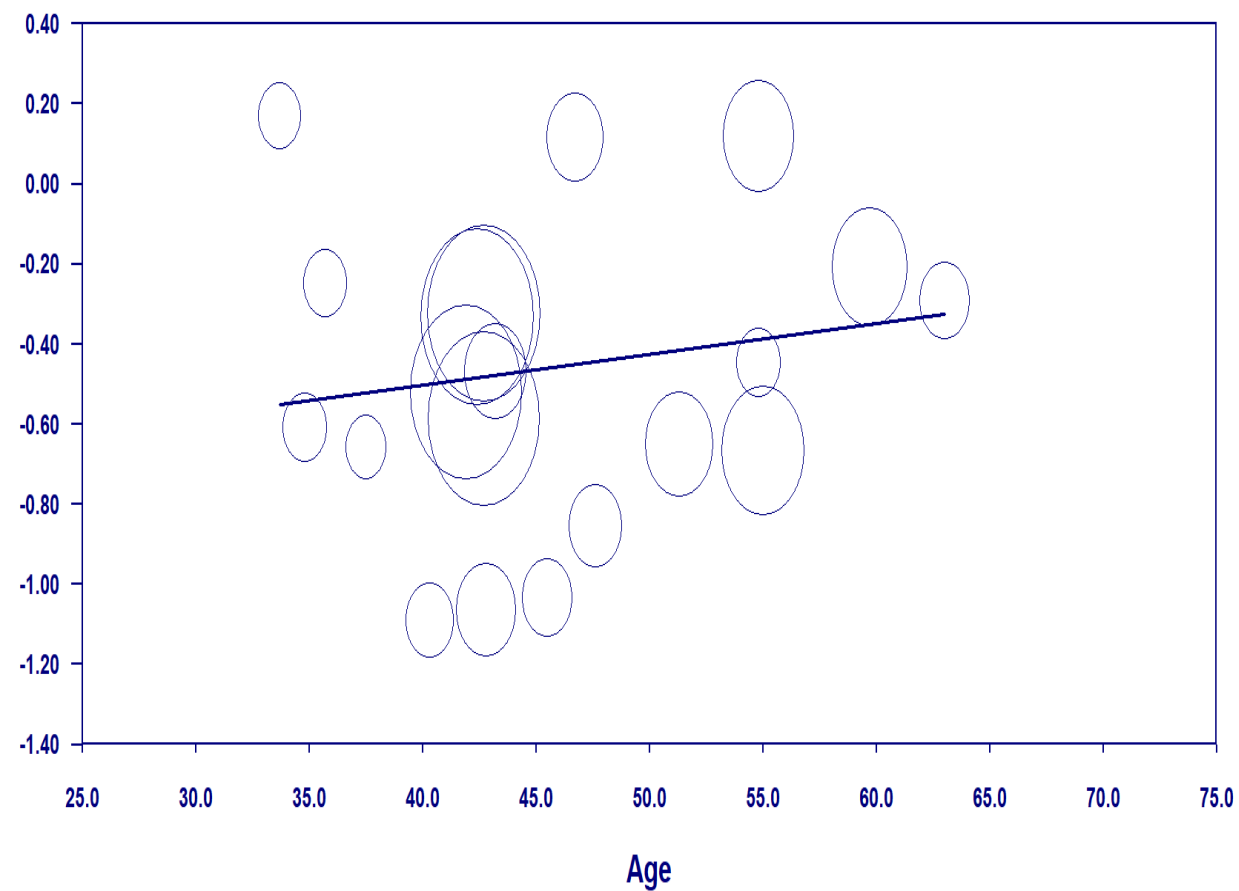

Supplement: Supplementary file 1 [file jcm-10-04078-s001.zip › Figure s1.pdf]

**Figure S2.**

**(a)**

**Regression of Std diff in means on BMI**

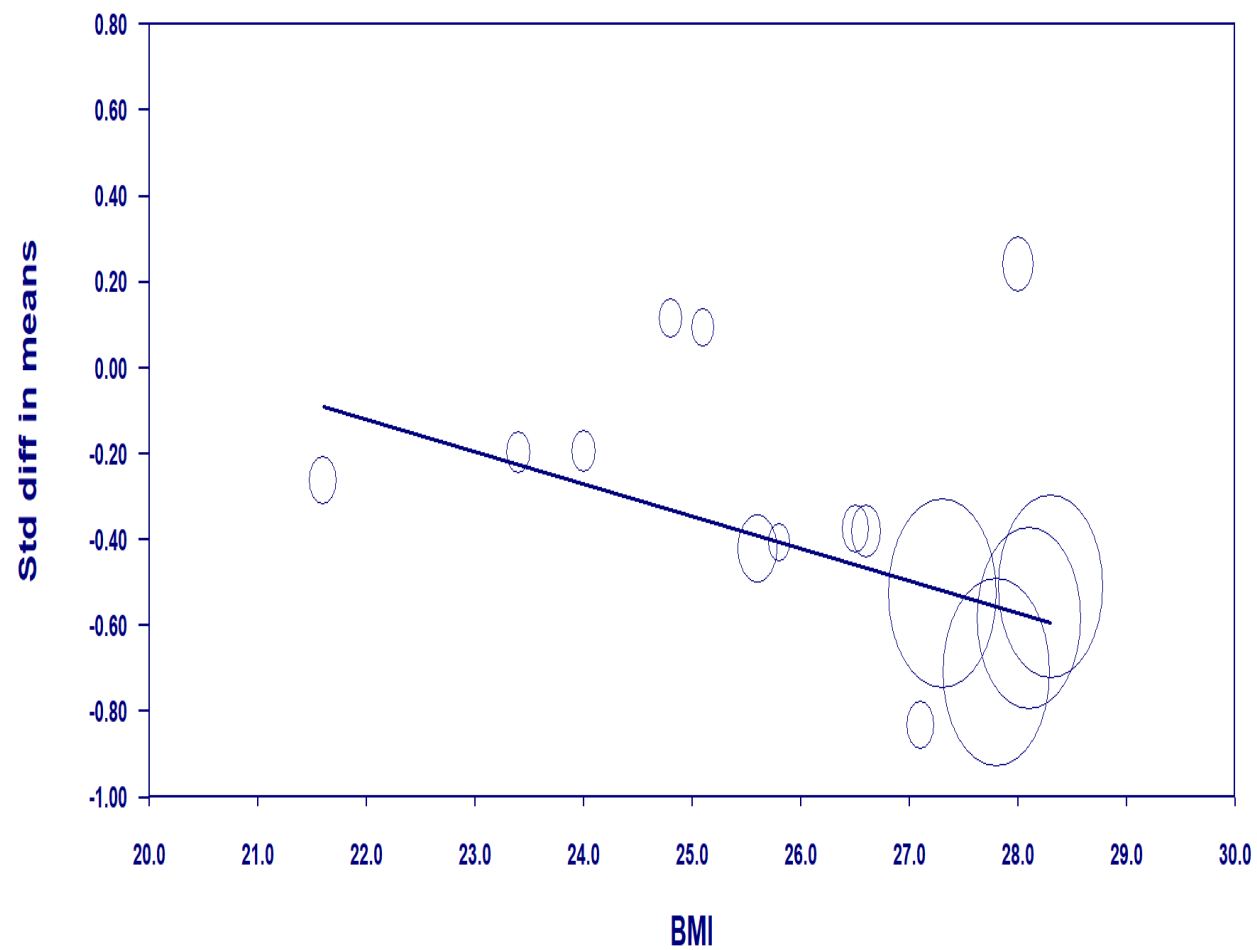

**(b)**

**Regression of Std diff in means on Age**

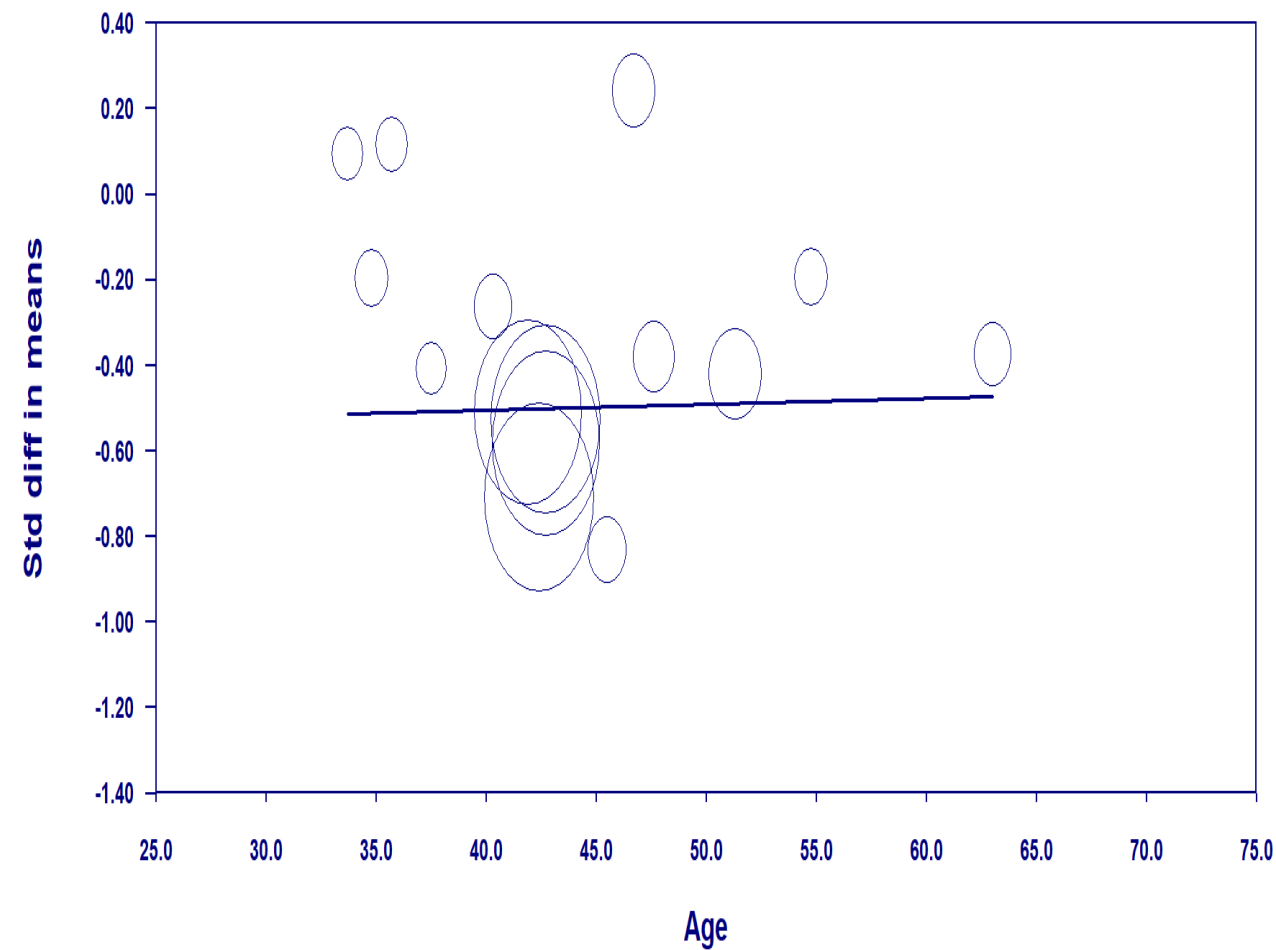

Supplement: Supplementary file 1 [file jcm-10-04078-s001.zip › Figure s2.pdf]

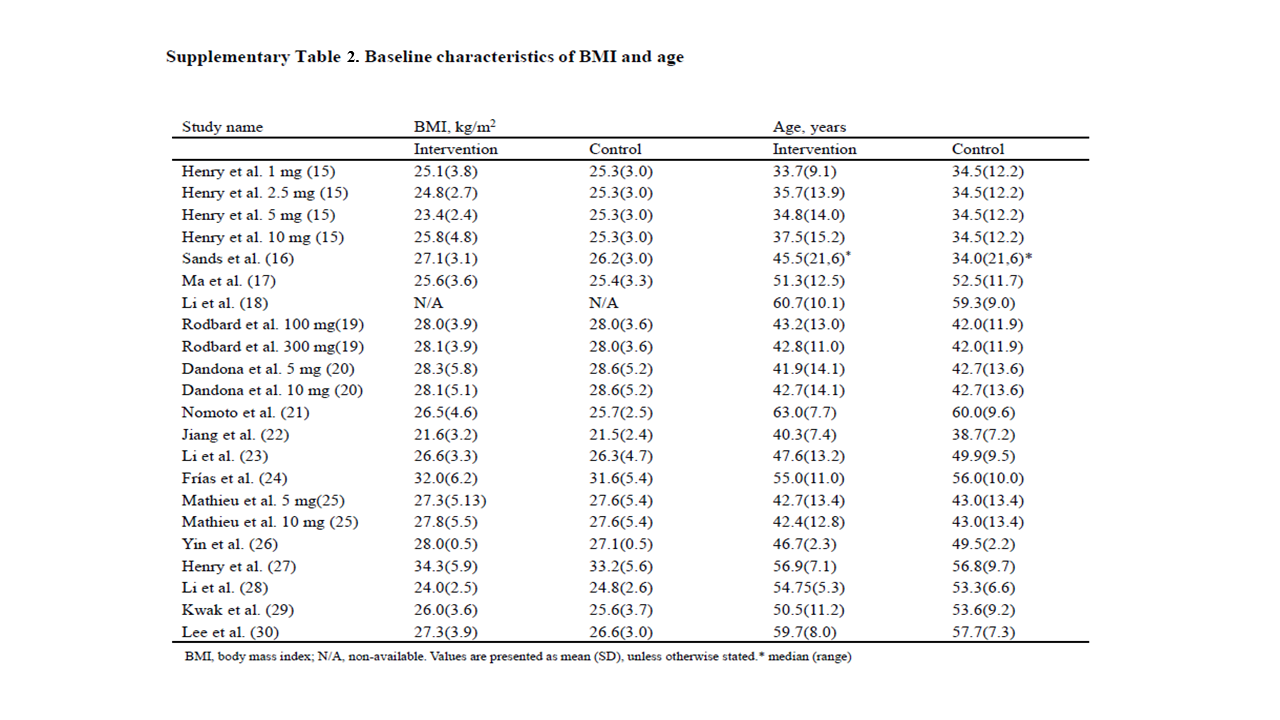

Supplement: Supplementary file 1 [file jcm-10-04078-s001.zip › Supplementary table 2.tiff]
